# Chemical Promoter Performance for CO_2_ Hydrate Growth: A Molecular Perspective

**DOI:** 10.1021/acs.energyfuels.3c00472

**Published:** 2023-04-10

**Authors:** Anh Phan, Alberto Striolo

**Affiliations:** † School of Chemistry and Chemical Engineering, Faculty of Engineering and Physical Sciences, University of Surrey, Guildford, Surrey GU2 7XH, U.K.; ‡ Department of Chemical Engineering, University College London, London WC1E 7JE, U.K.; § School of Chemical, Biological and Materials Engineering, University of Oklahoma, Norman, Oklahoma 73019, United States

## Abstract

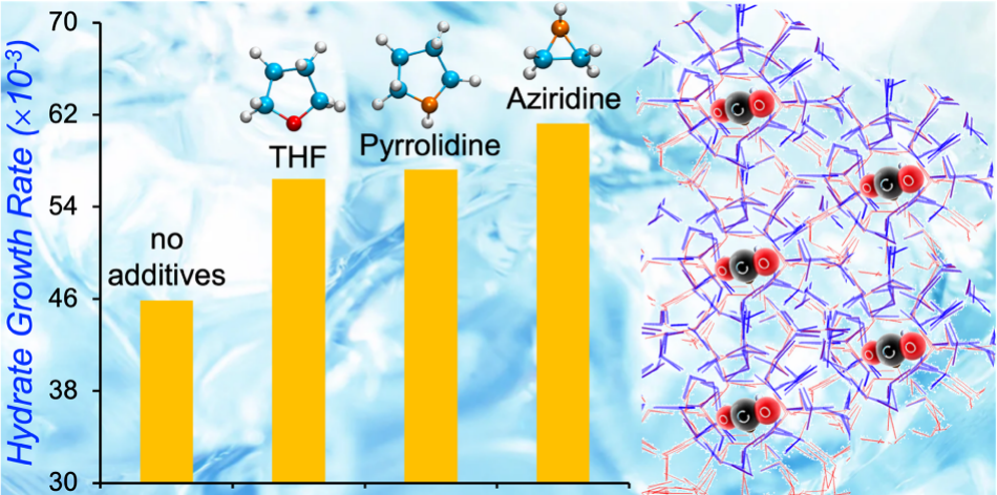

Carbon dioxide (CO_2_) hydrates, which contain a relatively large amount of captured CO_2_ (almost 30 wt % of CO_2_ with the balance being water), represent a promising CO_2_ sequestration option for climate change mitigation. To facilitate CO_2_ storage via hydrates, using chemical additives during hydrate formation might help to expedite formation/growth rates, provided the additives do not reduce the storage capacity. Implementing atomistic molecular dynamics, we study the impact of aziridine, pyrrolidine, and tetrahydrofuran (THF) on the kinetics of CO_2_ hydrate growth/dissociation. Our simulations are validated via reproducing experimental data for CO_2_ and CO_2_ + THF hydrates at selected operating conditions. The simulated results show that both aziridine and pyrrolidine could perform as competent thermodynamic and kinetic promoters. Furthermore, aziridine seems to exceed pyrrolidine and THF in expediting the CO_2_ hydrate growth rates under the same conditions. Our analysis unveils direct correlations between the kinetics of CO_2_ hydrate growth and a combination of the free energy barrier for desorption of CO_2_ from the hydrate surface and the binding free energy of chemical additives adsorbed at the growing hydrate substrate. The detailed thermodynamic analysis conducted in both hydrate and aqueous phases reveals molecular-level mechanisms by which CO_2_ hydrate promoters are active, which could help to enable the implementation of CO_2_ sequestration in hydrate-bearing reservoirs.

## Introduction

Climate change is one of the major challenges of our time. To mitigate excessive greenhouse gas emissions, countries and regions have proposed various plans.^1^ The Kyoto Protocol^2^ and the Paris Agreement^3^ are the two best examples of cooperation among humans. Different mitigation strategies have been mapped, which necessitate various combinations of reduced energy use, low-carbon energy supplies, and carbon dioxide (CO_2_) removal. Although the most effective strategies can aggravate socioeconomic inequalities between rich and poor countries, a risk to be alleviated, all approaches require long-term sequestration of CO_2_.^1^ According to the Intergovernmental Panel on Climate Change (IPCC) Fifth Assessment Report,^4^ CO_2_ storage is crucial to hold CO_2_ concentrations in the atmosphere below 450 ppm by 2100. It has been noted that the current lack of utilization of CO_2_ storage will intensify the expense of future CO_2_ storage operations by 138%, or more.^5^

Clathrate hydrates, discovered in 1811 by Sir Humphrey Davy,^6^ form because of a balance between guest–host (e.g., methane–water, CO_2_–water) dispersive interactions and hydrogen bonds among water molecules.^7^ Gas hydrates have attracted extensive industrial and scientific attention with the early stage of gas hydrate-related research focusing on flow assurance to avoid oil and gas pipeline blockage.^8−17^ Gas hydrate applications have expanded toward the water–energy–environment nexus including water desalination,^18,19^ gas separations,^20−22^ and gas storage.^23−25^ In addition, current research interests are focused on methods to alleviate global warming through long-term CO_2_ capture and storage.^25^ Even though CO_2_ storage, in the form of clathrate hydrates, is a promising technology, its practical implementation requires innovations to enable CO_2_-hydrate stability at near-ambient conditions, and to overcome the current kinetic limitations in hydrates’ decomposition due to well-known self-preservation effects.^26^ To date, a lack of knowledge of hydrate fundamentals has hindered attempts to make (near-) ambient storage a functional technology. Recent experimental and computational studies suggest that some promoters, such as tetrahydrofuran (THF), could stabilize CO_2_ hydrates at near-ambient conditions.^25,27,28^ Pyrrolidine is a cyclic secondary amine having a similar structure to THF whose five-membered ring contains four carbon atoms and one heteroatom. While the heteroatom in THF is oxygen, the one in pyrrolidine is nitrogen. Pyrrolidine not only has an analogous structure to THF, but its amine group is reminiscent of amino acids. Because some amino acids have been found to act as promoters for hydrate growth, pyrrolidine might act both as a thermodynamic and kinetic promoter.^29,30^ In fact, conducting experiments, Rangsunvigit et al.^29^ recently found that pyrrolidine has the potential to enhance the formation of methane (CH_4_) hydrates thermodynamically and kinetically. Having a N–H functional group embedded in a ring structure similar to pyrrolidine, aziridine—a three-membered nitrogen-containing heterocyclic compound—could also be a promising potential candidate for facilitating CO_2_ hydrate formation/growth. Since initially described by Gabriel in 1888,^31^ aziridine has attracted chemists due to its biological properties, mainly as an antitumor agent, or by its bond strain that makes it an effective prototype of more complex molecules.^32,33^ At present, there are no microscopic-level studies on aziridine or pyrrolidine used as promoters for CO_2_ hydrates.

Employing atomistic molecular dynamics (MD) simulations, we probe here the impact of aziridine, pyrrolidine, and THF, at various operating conditions, on CO_2_ hydrate growth/dissociation processes. We report CO_2_ hydrate growth/dissociation rates and conduct thermodynamic analysis upon the interactions between these chemical additives, CO_2_, and water molecules in the hydrate and aqueous phases. The simulation results are consistent with the experimental observations as interpreted in the literature; in addition, the insights available from the simulated trajectories identify the molecular mechanisms that could be prompted to advance the technologies for the implementation of CO_2_ capture and sequestration in hydrates. In the remainder of the manuscript, we first present the methodologies used for this study, then discuss the results, and conclude by summarizing the important observations and their practical implications.

## Simulation Methodology

### Model Setup

We implemented the “direct coexistence method,” which is the common computational approach to examine hydrate growth/dissociation within the aqueous phase.^34−36^ Thus, liquid–hydrate two-phase models were built to study the growth/dissociation of CO_2_ hydrates. It is known that, thermodynamically, structure sI is stable for CO_2_ guest molecules;^7^ hence, we employed a unit cell of sI CO_2_ hydrates^37^ to construct the solid substrate for CO_2_ hydrates. The sI CO_2_ hydrate unit cell was replicated four times in all directions (4.812 × 4.812 × 4.812 nm^3^) to generate the hydrate slab. The hydrate slab was surrounded by water and CO_2_ molecules, yielding a simulation box length of 4.812, 4.812, and 16 nm in the *X*-, *Y*-, and *Z*-directions, respectively. Applying periodic boundary conditions in three directions, the hydrate substrate model is effectively infinite along the *X* and *Y* directions.

**Figure 1:**
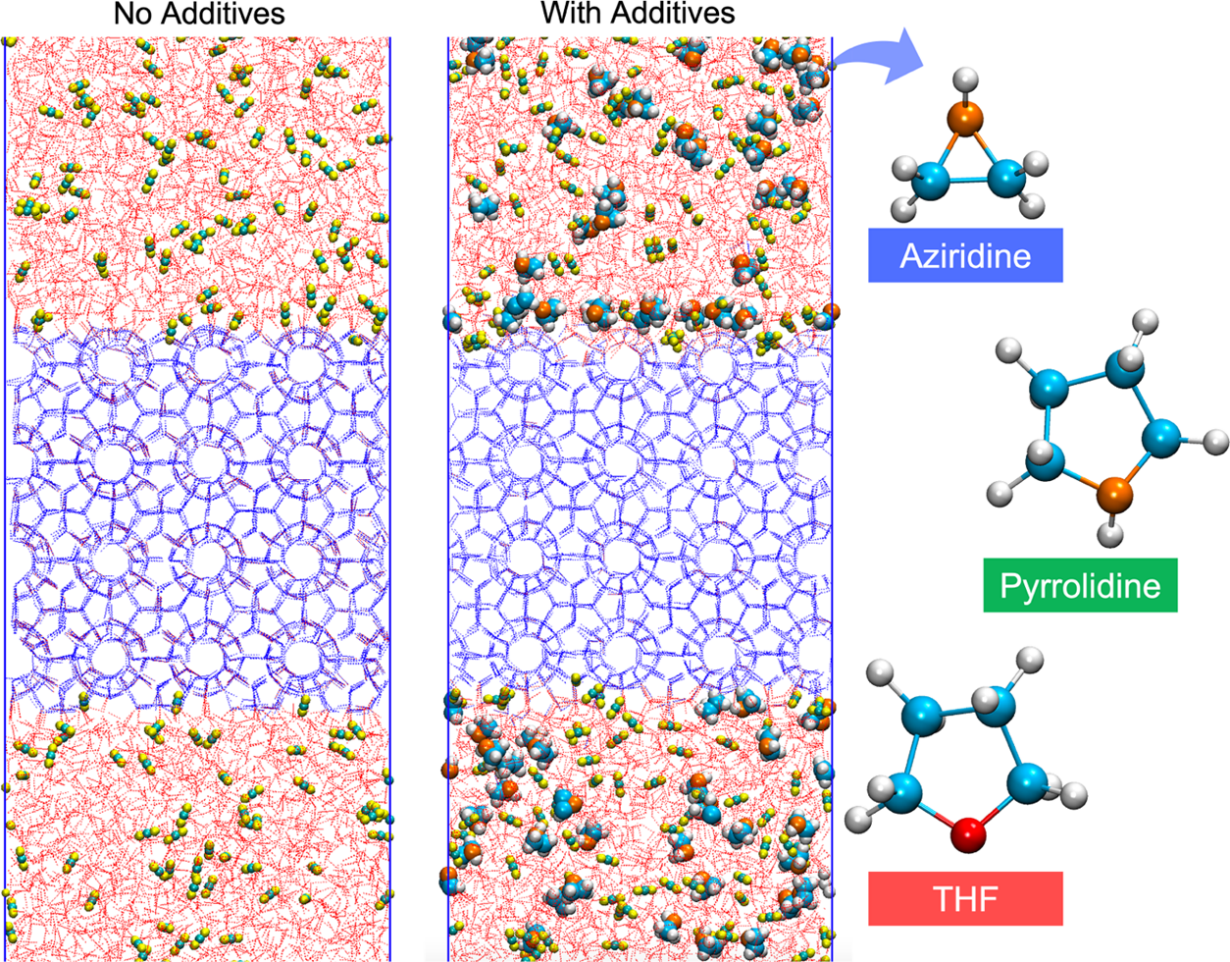
Representative simulation snapshots for the configurations for systems composed of solid CO_2_ hydrate substrate, water, and CO_2_ without (left) and with (right) chemical additives, e.g., aziridine, pyrrolidine, and THF molecules in the aqueous phase. Blue and red dotted lines symbolize water molecules in the hydrate and aqueous phases, respectively. Cyan and yellow spheres symbolize CO_2_ carbon and oxygen atoms, respectively. Orange, red, white, and blue spheres represent nitrogen, oxygen, hydrogen, and carbon atoms in chemical additive molecules, respectively.

Four systems are considered: CO_2_ hydrates without and with chemical additives, e.g., aziridine, pyrrolidine, and THF, as illustrated in the left and right panels of Figure 1, respectively. The number of chemical additives and CO_2_ molecules inserted into the bulk aqueous phase was 100 and 240, yielding the initial mole fractions of ∼1.42 and ca. 3.27–3.45%, respectively. The mole fraction of the chemical additives (∼1.42%) was chosen to be consistent with experimental observations reporting no large differences in the dissociation boundaries in the range of 3–6 mol % of THF for THF + CO_2_ hydrates.^38,39^ It should be noted that, because THF is a thermodynamic stabilizer for CO_2_ hydrates, for all THF concentrations considered here, the dissociation conditions of the THF + CO_2_ hydrates were shifted to lower pressures and higher temperatures compared to those of pure CO_2_ hydrates.^38,39^ The initial mole fractions of CO_2_ (∼3.27 to 3.45%) correspond to the solubility of CO_2_ in bulk aqueous phase at the chosen conditions.^25^

### Force Fields

Water molecules were simulated by the TIP4P/Ice model, which has been proven successful to study hydrate nucleation and growth^40,41^ and to describe the properties of liquid state at ambient conditions.^42^ CO_2_ was modeled using the EPM2 force field,^43^ which is appropriate for quantifying the growth/dissociation mechanisms of CO_2_ gas hydrates.^25,34,44^ Aziridine, pyrrolidine, and THF were modeled employing the General AMBER Force Field.^45,46^ All nonbonded interactions were described by electrostatic and dispersion forces. The Coulombic potential was used to characterize electrostatic interactions, with the particle–particle particle–mesh (PPPM) method^47^ for treating long-range corrections. The 12–6 Lennard-Jones (LJ) potentials were used to model dispersive interactions. The Lorentz–Berthelot combining rules were employed to describe the LJ parameters for unlike interactions from the values of like components.^48^ The cutoff distance of 14 Å was used for all interactions.

### Implementation

Equilibrium MD simulations were carried out using the package GROMACS.^49^ Initially, we performed an energy minimization protocol to relax high-energy initial configurations using the steepest descent method.^49^ The simulations were then conducted in the NVT canonical ensemble for 250 ps to relax the initial configuration, while the hydrate layer was kept fixed at the chosen temperatures. Subsequently, the simulations were performed within the isothermal–isobaric ensemble (NPT) under conditions appropriate for CO_2_ hydrate growth/dissociation studies (*T* = 269.1, 279.1, 284.1, and 289.1 K and *P* = 25.5 bar). The temperatures were kept constant by a Nosé–Hoover thermostat.^49^ We set 0.5 ps as the time constant for coupling between the system and the thermostat, which guarantees to remove latent heat from the growing hydrate surface rather quickly.^50^ The Parrinello–Rahman barostat^49^ was used to maintain the pressure at 25.5 bar, with the pressure coupling applied only along the *Z* direction of the simulation box (perpendicular to the hydrate–liquid interface). Hence, the simulation box lengths in the *X* and *Y* directions were maintained constant, which maintains the crystalline structure of the hydrate particle, and the hydrate surface area was kept the same for all systems. The leapfrog algorithm with 1.0 fs time step was used to integrate the equations of motion.^49^ All molecules in the system, including water and CO_2_ molecules within the hydrate surface, were allowed to move during the NPT simulations. We conducted each NPT simulation for 500 ns. To quantify the uncertainty in our results, we performed two additional simulation runs for all systems with the same hydrate composition at various temperatures.

### Potential of Mean Force (PMF) Calculations

**Figure 2:**
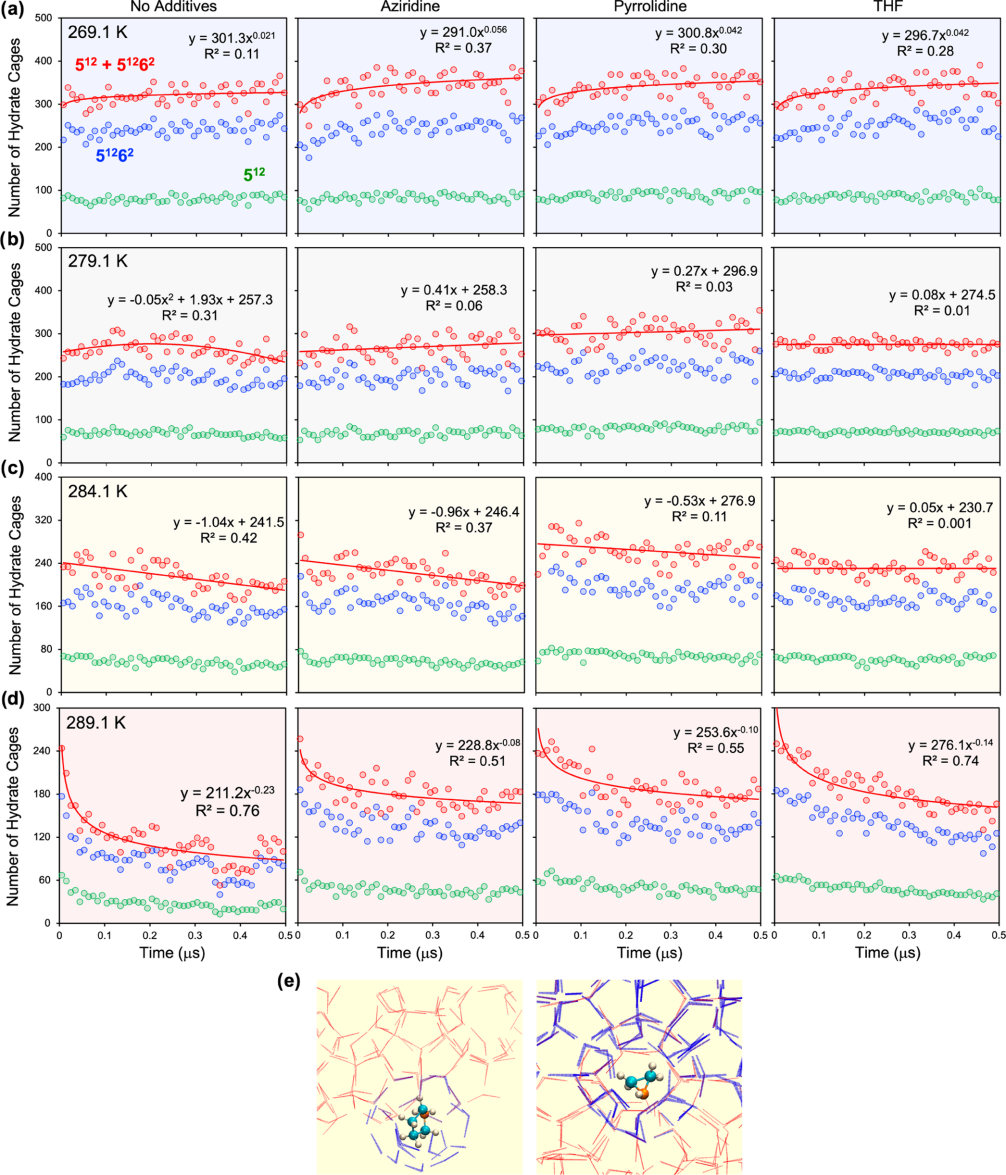
Number of various types of hydrate cages, e.g., 12-hedron (5^12^, green) and 14-hedron (5^12^6^2^, blue), and total number of both cages (5^12^ + 5^12^6^2^, red), as a function of time for systems composed of the sI CO_2_ hydrate without additives, in the presence of aziridine, pyrrolidine, or THF at various temperatures, such as 269.1 K (a), 279.1 K (b), 284.1 K (c), and 289.1 K (d). (e) Representative simulation snapshots of a 5^12^6^4^-cage filled with pyrrolidine and a 5^12^6^2^-cage holding aziridine. The results were obtained at 269.1 K during 500 ns of simulations.

We employed the umbrella sampling (US) technique^49^ to compute the PMF profiles experienced by one CO_2_ molecule migrating from the hydrate substrate to the bulk aqueous phase across the growing hydrates. The configurations for systems at the beginning of hydrate growth simulations at 269.1 K were used as the initial configurations for conducting US calculations. The outermost layer of the hydrate slab comprises filled small cages (5^12^ cages) and open large cages (5^12^6^2^ cages) on its surface. One CO_2_ molecule in such an open large cage at the outermost layer of the hydrate substrate was tagged. The tagged CO_2_ molecule was allowed to oscillate around given *Z* positions along a trajectory perpendicular to the hydrate surface using a harmonic tether with a force constant of 2000 kJ/(mol·nm^2^),^49^ while the movements along the *X* and *Y* directions were not allowed. We applied a harmonic restraint force constant of 2000 kJ/mol/nm^49^ on the rest of the carbon atoms of the CO_2_ molecules in the hydrate phase to tether them to their initial positions. Before conducting US simulations, we first performed NVT simulations for 1 ns to relax the initial configuration and then NPT simulations at 269.1 K and 25.5 bar. Because CO_2_ hydrates grow significantly within the first 100 ns of simulations (as shown, e.g., in Figure 2a), we conducted the NPT simulations for 40 ns before US simulations to examine the interactions between chemical additives, CO_2_, and water molecules during the growth. Once the 40 ns NPT simulations were completed, we constructed the PMF profiles as a function of the *Z* distance between the tagged and a reference CO_2_ molecule in the hydrate substrate (distance *l*); both the tagged and reference CO_2_ molecules are allowed to rotate while being tethered to its initial positions with a force constant of 2000 kJ/(mol·nm^2^). The reference CO_2_ molecule in the hydrate substrate is located at *Z* = 9.6 nm. Details regarding the algorithm are described elsewhere.^51^ Thirty-three independent NVT simulations were conducted to produce the corresponding 33 windows for each PMF profile, with two adjacent windows separated by 0.1 nm to achieve overlaps of the correspondent density profiles. The PMF profiles were reconstructed implementing the weighted histogram analysis method (WHAM) technique.^52^ Errors are estimated from bootstrap analysis implemented in GROMACS.^49^ We employed this US procedure to construct PMF profiles for one chemical additive molecule (e.g., aziridine, pyrrolidine, and THF) as it travels from the growing hydrate surface to the bulk aqueous phase.

## Results and Discussion

### Hydrate Growth Kinetics

To quantify hydrate structural growth, the cage occupancy and cage type were assessed using the topological-algorithm-assisted cage identification.^53^ In Figure 2, we report the number of various types of hydrate cages, e.g., 12-hedron (5^12^, green) and 14-hedron (5^12^6^2^, blue), and the total number of these two types of cages (5^12^ + 5^12^6^2^, red), as a function of simulation time for systems composed of sI hydrate without additives, in the presence of aziridine, pyrrolidine, or THF at various temperatures, e.g., 269.1 K (a), 279.1 K (b), 284.1 K (c), and 289.1 (d) K. The system conditions were chosen, based on the prior work,^25^ to prevent the integration of the chemical additives into the hydrate cages. Analysis of the simulation trajectories confirms that additive molecules were barely trapped in the hydrates at the end of the simulation. It is concluded that, at the conditions chosen here, the additives do not compromise the storage capacity of the hydrates. The number of hydrate cages grows exponentially at 269.1 K in all systems considered (see Figure 2a). Fitting the results with an exponential curve, we extract cage growth rates. Our analysis revealed that the values of fitting exponents increase in the following order: no additives (21 × 10^–3^) < THF ∼ pyrrolidine (42 × 10^–3^) < aziridine (56 × 10^–3^). This suggests that while pyrrolidine and THF show similar performance in enhancing the kinetics of CO_2_ hydrate formation/growth, aziridine could outperform both THF and pyrrolidine as an effective CO_2_ hydrate promoter at 269.1 K. Visual inspection of simulation snapshots for the systems at 269.1 K (see Figure 2e) shows that while pyrrolidine occupies 5^12^6^4^ hydrate cages forming the mixed CO_2_ + pyrrolidine sII hydrates, similar to results obtained for the mixed CO_2_ + THF sII hydrates,^25^ aziridine could be trapped in 5^12^6^2^ cages maintaining the CO_2_ + aziridine sI hydrate structure, possibly due to the smaller molecular size of aziridine. However, based on identification obtained via the cage identification algorithm,^53^ we point out that these cages are short-lived, with lifetimes of ∼10 ns. In fact, analysis of our simulation trajectories shows that the chemical additives promote the formation of hydrate cages but move further when the cage is filled by CO_2_.

In Figure 2b, the results show that for the system without chemical additives at 279.1 K, the CO_2_ hydrates grow, but they quickly reach a plateau, and then gradually dissociate as the simulations progress. When the chemical additives are present, the hydrates grow linearly at 279.1 K, with the growth rate increasing in the following order: THF < pyrrolidine < aziridine, suggesting that both aziridine and pyrrolidine could expedite the kinetics of CO_2_ hydrate growth more effectively than THF does. The results presented in Figure 2c show that at 284.1 K, the hydrates dissociate for the systems without chemical additives and even in the presence of aziridine and pyrrolidine; however, the hydrates still grow slowly when THF is present. When the temperature is further increased to 289.1 K, the CO_2_ hydrates simulated here dissociate following exponential trends even in the presence of THF (see Figure 2d). Fitting the results for the number of hydrate cages obtained at 289.1 K with exponential functions, we find that the values of fitting powers increase in the following order: no additives (−0.23) < THF (−0.14) < pyrrolidine ∼ aziridine (−0.1). These results suggest that the chemical additives help to delay the CO_2_ hydrate dissociation process in comparison to the case when no additives were used. Aziridine and pyrrolidine seem to be slightly more effective in impeding the dissociation of CO_2_ hydrates compared to THF.

Note that from experimental phase equilibrium data, the CO_2_ + THF hydrates are stable at temperatures <∼288.5 K at ∼1.42 mol % THF while pure CO_2_ hydrates are not stable at temperatures ≥279.1 K at 25.5 bar.^39,54^ This confirms that our simulated results reproduce accurately the thermodynamic stability of CO_2_ + THF and pure CO_2_ hydrates at the selected pressures and temperatures.^25^ Recently, Shirts et al.^55^ were able to predict temperature-mediated polymorphic transformations for 12 organic small-molecule systems using alchemical free energy calculations;^56^ this promising result suggests that alchemical free energy calculations could be a useful algorithm for investigating of thermodynamic stability of hydrates, and especially the transformation of hydrate structure during growth. We also found that both aziridine and pyrrolidine shift the dissociation boundary of pure CO_2_ hydrates to higher temperatures in the range between 279.1 and 284.1 K at 25.5 bar at ∼1.42 mol % aziridine/pyrrolidine. This means that these chemical additives have the potential to be thermodynamic promoters for the CO_2_ hydrates. However, neither aziridine nor pyrrolidine could alter the equilibrium curve as much as THF does. Rangsunvigit et al.^29^ recently conducted experiments to examine the morphology, thermodynamics, and kinetics of CH_4_ hydrates in the presence of 5.56 mol % pyrrolidine. They showed that even though pyrrolidine cannot be as effective as THF, it could move the phase equilibrium curve of pure sI CH_4_ hydrates to milder conditions, which means pyrrolidine could be a thermodynamic promoter for CH_4_ hydrates.^29^ Conventional wisdom suggests that thermodynamic promoters are likely to function more effectively if they themselves can form hydrates without any support from other guest gas molecules,^57^ which is the case for THF;^58^ on the contrary, pyrrolidine fails to form clathrate hydrates with host water but instead needs to associate with other guest molecules.^59^ Rangsunvigit et al.^29^ also examined the effects due to subcooling on the CH_4_ hydrate growth in the presence of pyrrolidine and THF. They reported that the average final CH_4_ uptake with different promoters at the same subcooling degree is comparable to the one obtained by conducting the experiments at the same operating conditions (i.e., slightly different subcooling).^29^ This suggests that the investigation of the kinetics of CO_2_ hydrate growth/dissociation without and with the chemical additives at the same subcooling degree would provide similar results to those reported here.

In addition, Rangsunvigit et al. also found that pyrrolidine could be a kinetic promoter for CH_4_ hydrate formation/growth.^29^ Specifically, under certain conditions, e.g., *P* = 80 bar and *T* = 285.2 K, the presence of 5.56 mol % of pyrrolidine shortens the induction time of CH_4_ hydrates formation in comparison to THF.^29^ Investigating the dissociation processes of CH_4_ + THF and CH_4_ + pyrrolidine hydrates, the experimental results showed that the CH_4_ + THF hydrates dissociated faster than the CH_4_ + pyrrolidine hydrates under the same driving force.^29^ These experimental observations could be attributed to the fact that pyrrolidine is able to form hydrogen bonds with water through its nitrogen and hydrogen atoms,^60^ while THF interacts with water as a hydrogen-bond acceptor through its oxygen atom only.^61^ Our simulation results also show that pyrrolidine increases the CO_2_ hydrate growth rates more effectively than THF does at 279.1 K and 25.5 bar. We also observe that pyrrolidine helps to slow down the CO_2_ hydrate dissociation somewhat more effectively than THF at 289.1 K. Qualitatively consistent with literature experiments,^29^ these results suggest that pyrrolidine can also perform as a thermodynamic and kinetic promoter for the CO_2_ hydrates. However, because the experimental data just referred to were obtained for CH_4_ hydrates, further experimental studies for CO_2_ hydrate formation in the presence of pyrrolidine should be conducted to verify our observations.

Our computational findings suggest that both aziridine and pyrrolidine could perform as efficient thermodynamic and kinetic promoters for CO_2_ hydrate growth. Furthermore, aziridine exceeds both pyrrolidine and THF in facilitating the kinetics of CO_2_ hydrate formation/growth under the same conditions, e.g., *T* = 269.1 K (see Figure 2a). One fundamental question emerges from the quantitative analysis for CO_2_ hydrate growth at 269.1 K: what are the molecular mechanisms by which these chemical additives affect CO_2_ hydrate growth? To tackle this question, we perform detailed thermodynamic calculations because an interplay of chemical additives, CO_2_, and water molecules in the bulk and hydrate phases seems to promote different CO_2_ hydrate growth rates.

**Figure 3:**
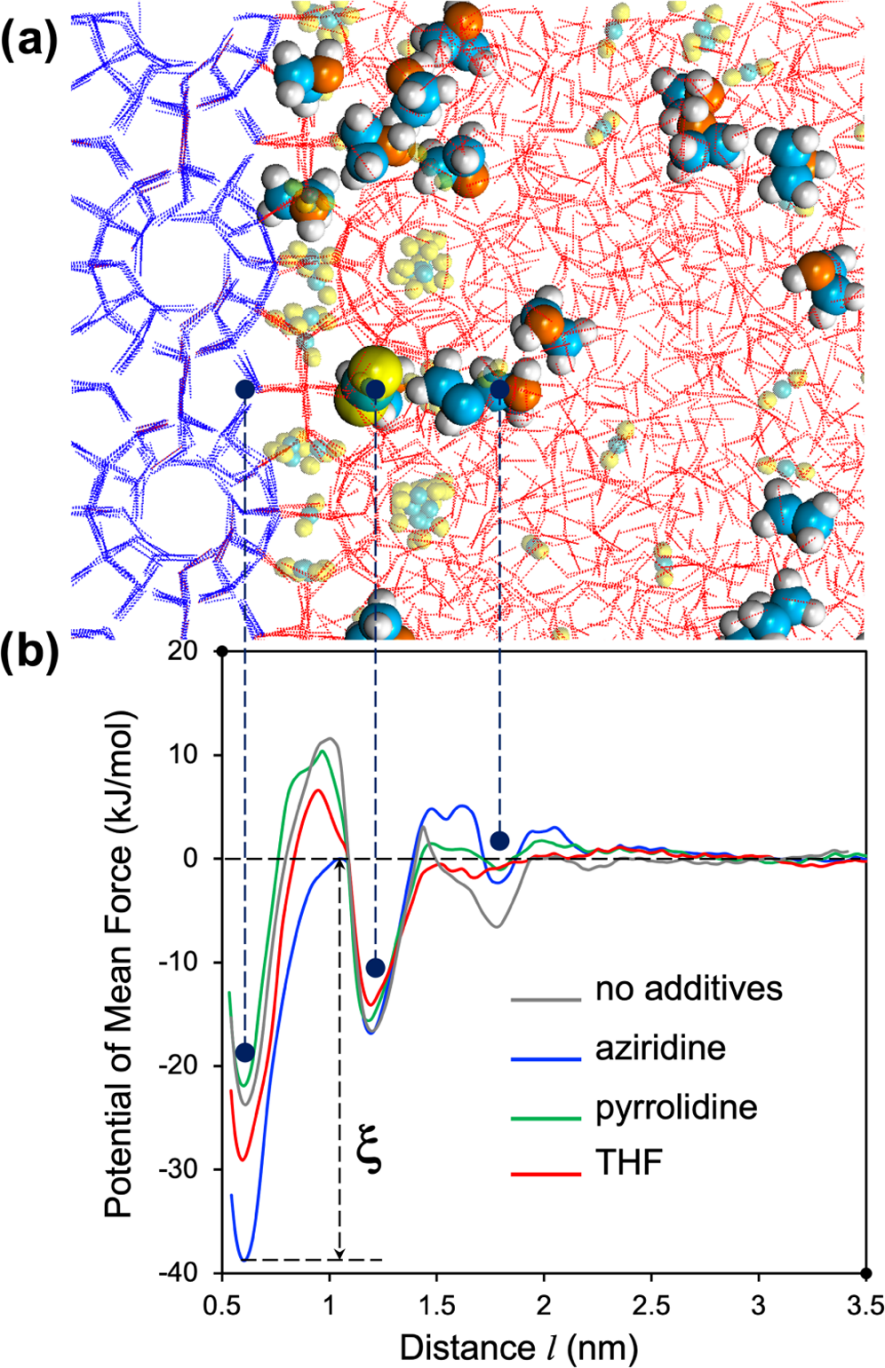
(a) Representative simulation snapshot illustrating the configuration for one CO_2_ molecule traversing the growing hydrates to the bulk aqueous phase along the *Z* direction (perpendicular to the hydrate surface). (b) PMF profiles obtained for the tagged CO_2_ molecule as it moves from the hydrate surface to the bulk aqueous phase across the growing hydrate layer. Results were obtained for systems composed of the sI CO_2_ hydrate without chemical additives (gray), in the presence of 1.42 mol % aziridine (blue), pyrrolidine (green), or THF (red) at 269.1 K.

To quantify whether interactions between guest CO_2_ molecules with the hydrates in the presence of chemical additives (e.g., aziridine, pyrrolidine, and THF) relate to the hydrate growth rates, we calculated PMF profiles as experienced by one CO_2_ molecule moving from the hydrate surface to the bulk aqueous phase across the growing hydrates (see illustration in Figure 3a). In Figure 3b, we present the resultant PMF profiles. Results were obtained for systems composed of sI CO_2_ hydrate without chemical additives (gray), in the presence of 1.42 mol % aziridine (blue), pyrrolidine (green), or THF (red) at 269.1 K. In general, the results show an attractive free energy well when the tagged CO_2_ is placed in an open 5^12^6^2^ cage at the outermost layer of the hydrate surface. As the distance *l* increases, the PMF profiles show one moderate repulsive barrier at intermediate distances *l*, a subsequent minimum, and a small repulsive barrier as *l* increases. The results also show that there are no long-range interactions between the CO_2_ guest molecules and the hydrate surface, which is qualitatively consistent with the results reported by Yagasaki et al.^62^ (Note that macroscopic effects such as concentration gradient might exist; these effects are not accounted for in our PMF analysis). To quantify the molecular mechanisms triggered by the presence of chemical additives on the PMF profiles, we calculated the free energy barrier for the desorption of CO_2_ from the hydrate surface, ξ, highlighted in Figure 3b. The results show that the presence of chemical additives alters ξ in the following order: pyrrolidine (32.3 ± 0.8 kJ/mol) < no additives (35.3 ± 0.8 kJ/mol) ∼ THF (35.6 ± 0.8 kJ/mol) < aziridine (38.7 ± 0.9 kJ/mol). One would expect that the more difficult it is for CO_2_ molecules to desorb from the growing hydrate surface (higher ξ), the faster the CO_2_ hydrate growth rates could be.^63^ However, the values for ξ do not correlate with the corresponding CO_2_ hydrate growth rates at 269.1 K reported in Figure 2a.

Previous experimental and computational studies indicate that THF significantly facilitates the diffusivity of CO_2_ at hydrate–aqueous interfaces,^25,64^ probably because the stronger THF–water hydrogen-bonding interactions weaken the CO_2_–water interactions, enabling CO_2_ to travel larger distances. This enhanced diffusivity might contribute to the speed-up of CO_2_ hydrate formation/growth.^25,64^ Because of the significant contribution of synergistic interactions of CO_2_–host water–chemical additives at interfaces in facilitating CO_2_ guest transports,^64^ which could lead to expedite CO_2_ hydrate growth, we computed the binding free energy (Δ*G*_bind_) for one chemical additive molecule at the growing hydrate–aqueous interface as the difference of free energies in the bound and unbound states^16^
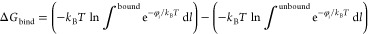
1where φ_*i*_ is the PMF value associated with the *i*th bin along the path (with distance *l*) where one chemical additive molecule travels from the growing hydrate surface to the bulk aqueous phase.

**Figure 4:**
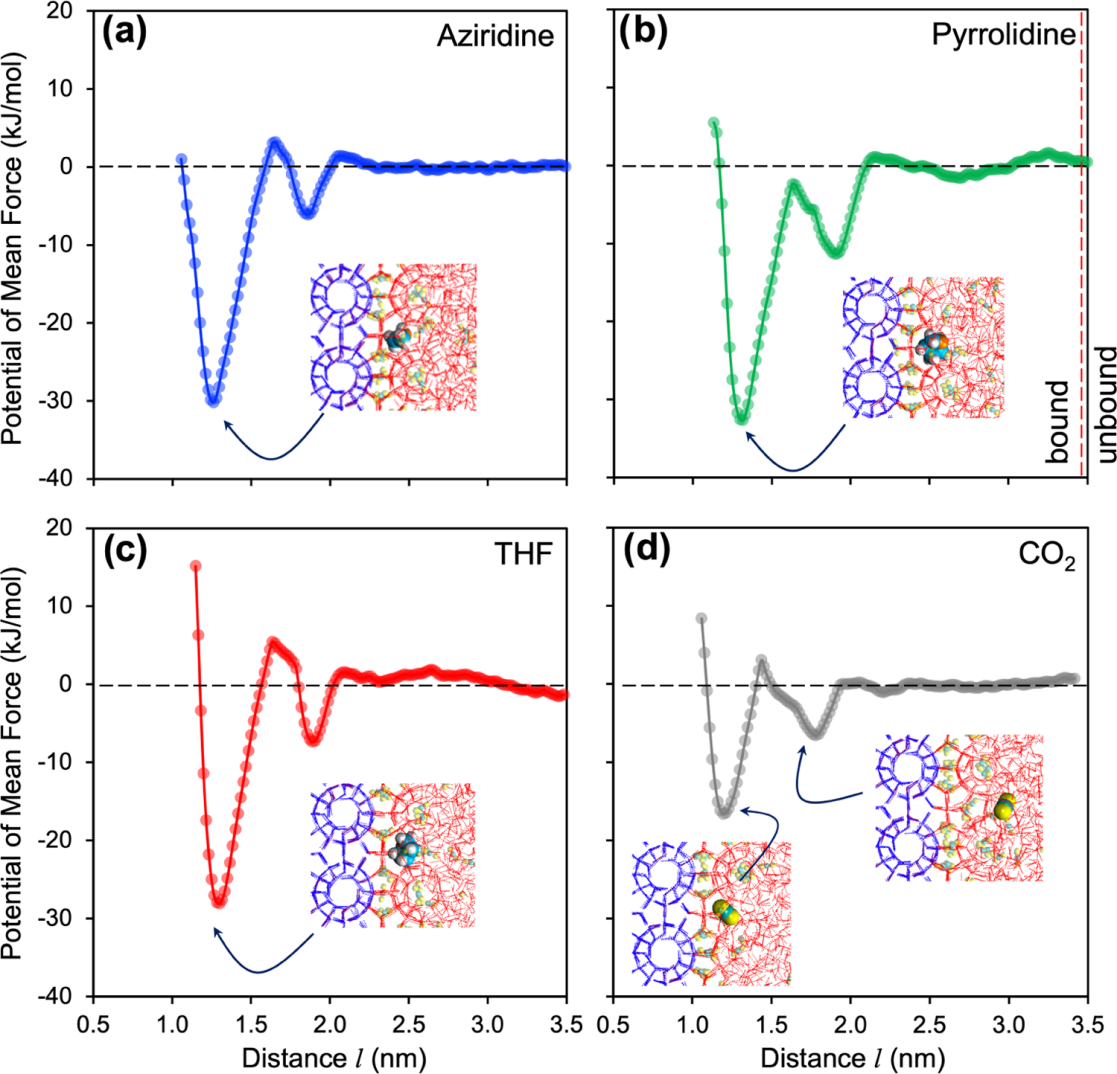
Potential of mean force (PMF) profiles obtained for one aziridine (a), pyrrolidine (b), or THF (c) molecule as it travels from the growing hydrate surface to the bulk aqueous phase. Results were obtained for the systems in which no chemical additives are present in the bulk aqueous phase (except for the tagged one) at 269.1 K. The PMF profile obtained for the tagged CO_2_ (d) was also shown for comparison.

To obtain the values of φ_*i*_ as shown in eq 1, we construct PMF profiles for one chemical additive (e.g., aziridine, pyrrolidine, and THF) molecule as it travels from the growing hydrate surface to the bulk aqueous phase. Note that the computation of binding free energy requires the inclusion of only one adsorbate molecule of interest at solid–liquid interfaces.^16,62,65−67^ Hence, in these PMF calculations, no chemical additives are present in the bulk aqueous phase, except for the one considered for the calculations. The additive molecule considered for the calculations is allowed to rotate at a given *Z* position to which its center of mass is tethered. We employed the simulation procedures analogous to those used in the previous section. In Figure 4, the results for PMF profiles are shown for the tagged aziridine (a), pyrrolidine (b), and THF (c). We also present the PMF profile for the tagged CO_2_ molecule (d) for comparison. The results show an effective strongly attractive interaction when the tagged chemical additive/CO_2_ molecule is located at the growing hydrate surface (next to the filled 5^12^6^2^ cage at the outermost layer of the hydrate substrate considered in the PMF calculations for the tagged CO_2_ molecule). As the distance *l* increases, the PMFs first show one extremely small repulsive barrier, and then a subsequent small minimum.

Employing eq 1 for the PMF profiles shown in Figure 4, we obtain the values of Δ*G*_bind_ for the systems considered in the following order: CO_2_ (10.5 ± 0.2 kJ/mol) < THF (20.8 ± 0.5 kJ/mol) < aziridine (22.6 ± 0.5 kJ/mol) < pyrrolidine (25.0 ± 0.6 kJ/mol). These results show that CO_2_ is less strongly adsorbed at the growing hydrate substrate than all of the chemical additives considered here, probably because of weakened CO_2_–water hydrogen bonding. The preferential adsorption of aziridine and pyrrolidine over THF at the growing hydrate surface is likely due to the fact that both aziridine and pyrrolidine are heterocyclic compounds having N–H functional groups, which could interact with water as both hydrogen-bond donor and acceptor simultaneously.^60^ Our simulation results suggest no correlation between the Δ*G*_bind_ and the corresponding CO_2_ hydrate growth rates at 269.1 K (reported in Figure 2a).

**Figure 5:**
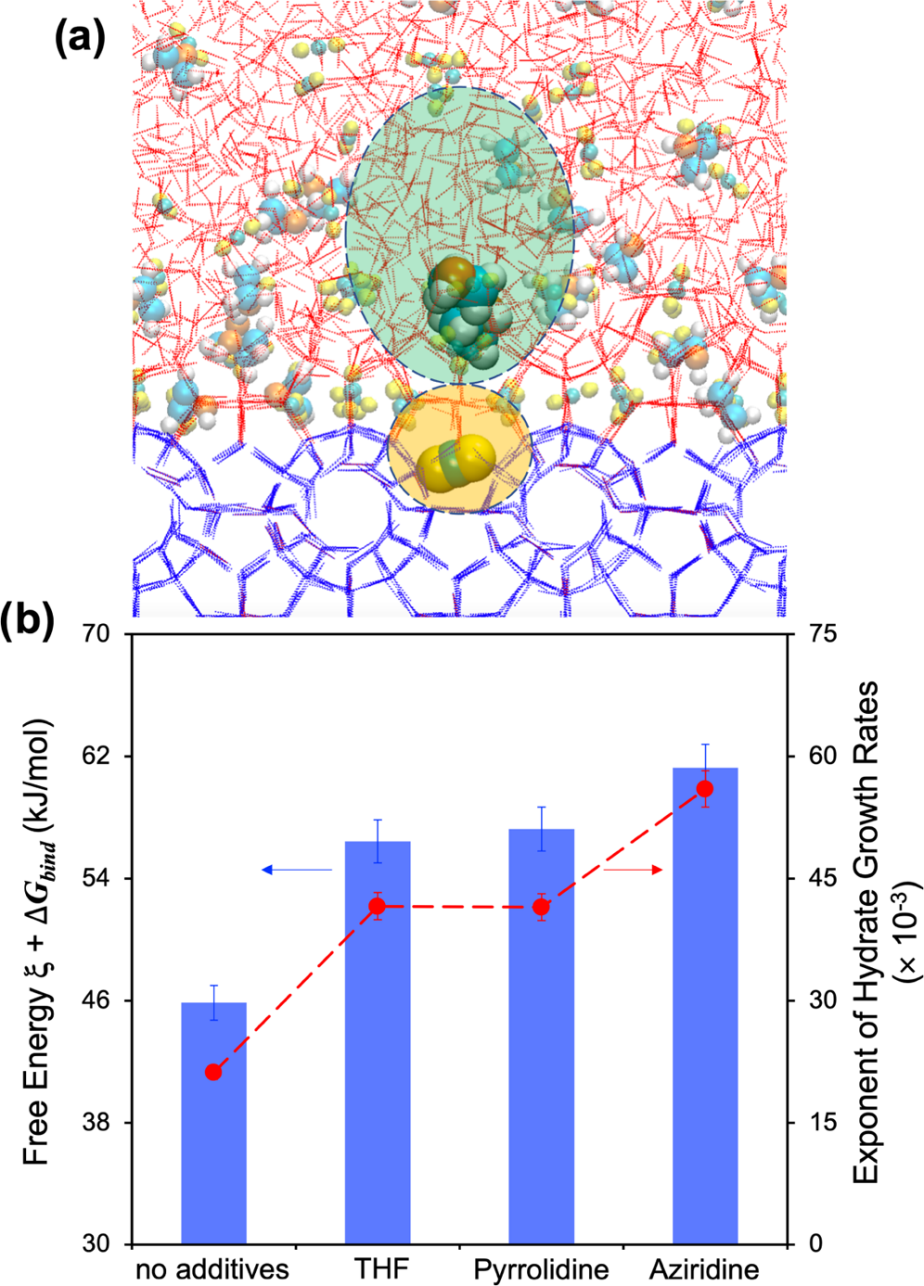
(a) Representative simulation snapshot illustrating the configuration for one CO_2_ molecule occupying one of the 5^12^6^2^ cages at the outermost layer of the hydrate substrate and one chemical additive adsorbed on the growing hydrate layer. (b) Sum of the free energy barrier for desorption of CO_2_ from the hydrate surface (ξ) and the binding free energy of chemical additives at the growing hydrate substrate (Δ*G*_bind_) (bars) with the corresponding exponent of hydrate cage growth rates (filled circles).

However, by combining the free energy barrier for desorption of CO_2_ from the hydrate surface (ξ) and the binding free energy of chemical additives adsorbed at the growing hydrate substrate (Δ*G*_bind_) (see the representative snapshot shown in Figure 5a), we observe a direct correlation for all systems considered: i.e., the higher the value of ξ + Δ*G*_bind_ (bars), the higher the CO_2_ hydrate growth rates at 269.1 K and 25.5 bar (filled circles) (see Figure 5b). This suggests that quantifying ξ for CO_2_ molecules occupying the hydrate cages in the outermost layer of the hydrate substrate and Δ*G*_bind_ for chemical additives adsorbed on the growing hydrates could be useful for predicting changes in the kinetics of CO_2_ hydrate formation/growth. More comprehensive studies should be carried out for a variety of chemical additives, possibly led by a design of experimental setup, to determine whether this correlation is general.

## Conclusions

Employing classical molecular dynamics simulations, we investigated CO_2_ hydrate growth/dissociation processes in the presence of nitrogen-containing heterocyclic compounds, e.g., aziridine and pyrrolidine, at the pressure of 25.5 bar and at temperatures 269.1, 279.1, 284.1, and 289.1 K. The results obtained for these chemical additives were also compared with those obtained in the presence of THF, an effective thermodynamic CO_2_ hydrate promoter. Using cage identification algorithms, our simulation results reproduce the experimental phase equilibrium data for CO_2_ + THF and pure CO_2_ hydrates at the selected pressure and temperatures; particularly, CO_2_ + THF hydrates are found to be stable at temperatures <∼288.5 K at ∼1.42 mol % THF, while pure CO_2_ hydrates are not stable at temperatures ≥279.1 K at 25.5 bar.

In addition, our simulation results show that both aziridine and pyrrolidine could perform as effective thermodynamic promoters for CO_2_ hydrate formation, although they cannot shift the coexistence curves as effectively as THF does. Indeed, according to our simulations, at 284.1 K, the CO_2_ hydrates dissociate in the presence of aziridine and pyrrolidine, while the hydrates grow when THF is present. Of note, at 269.1 K and 25.5 bar, the chemical additives considered scarcely adsorb within the hydrate cages. This implies that, under these conditions, the storage capacity of CO_2_ hydrates would not be compromised by the use of these additives. This study also suggests that aziridine and pyrrolidine could be kinetic promoters for CO_2_ hydrates, with aziridine outperforming pyrrolidine and THF in the enhancement of CO_2_ hydrate growth rates at 269.1 K and 25.5 bar.

Perhaps more significantly toward the possibility of identifying new effective chemical additives, this study reveals phenomenological correlations between the kinetics of CO_2_ hydrate growth and a combination of the free energy barrier for desorption of CO_2_ from the hydrate surface and the binding free energy of chemical additives adsorbed on the growing hydrates. Further computational studies should be conducted for other chemical additives, when possible with the aid of experimental observations, to evaluate whether this correlation is generalizable. Our results highlight the significance of molecular thermodynamics in unraveling the molecular mechanisms responsible for the chemical promoter performance in the implementation and optimization of CO_2_ capture and sequestration in clathrate hydrates.

## References

[ref1] Yu Y.-S.; Zhang X.; Liu J.-W.; Lee Y.; Li X.-S. Natural Gas Hydrate Resources and Hydrate Technologies: A Review and Analysis of the Associated Energy and Global Warming Challenges. Energy Environ. Sci. 2021, 14, 5611–5668. 10.1039/D1EE02093E.

[ref2] United Nations Framework Convention on Climate Change (UNFCCC). Kyoto Protocol Reference Manual on Accounting of Emissions and Assigned Amount, 2008.

[ref3] United Nations Framework Convention on Climate Change (UNFCCC). In Adoption of the Paris Agreement, 21st Conference of the Parties, Paris, 2015.

[ref4] Intergovernmental Panel on Climate Change (IPCC). AR5 Synthesis Report: Climate Change 2014, 2014.

[ref5] Al Baroudi H.; Awoyomi A.; Patchigolla K.; Jonnalagadda K.; Anthony E. J. A Review of Large-Scale Co2 Shipping and Marine Emissions Management for Carbon Capture, Utilisation and Storage. Appl. Energy 2021, 287, 116510–116551. 10.1016/j.apenergy.2021.116510.

[ref6] Koh C. A. Towards a Fundamental Understanding of Natural Gas Hydrates. Chem. Soc. Rev. 2002, 31, 157–167. 10.1039/b008672j. 12122641

[ref7] Dendy Sloan E.; Koh C. A. Clathrate Hydrates of Natural Gases, 3rd ed.; CRC Press - Taylor & Francis Group, 2007; p 752.

[ref8] Aman Z. M.; Koh C. A. Interfacial Phenomena in Gas Hydrate Systems. Chem. Soc. Rev. 2016, 45, 1678–1690. 10.1039/C5CS00791G. 26781172

[ref9] Striolo A. Clathrate Hydrates: Recent Advances on Ch4 and Co2 Hydrates, and Possible New Frontiers. Mol. Phys. 2019, 117, 3556–3568. 10.1080/00268976.2019.1646436.

[ref10] Striolo A.; Phan A.; Walsh M. R. Molecular Properties of Interfaces Relevant for Clathrate Hydrate Agglomeration. Curr. Opin. Chem. Eng. 2019, 25, 57–66. 10.1016/j.coche.2019.08.006.

[ref11] Naullage P. M.; Bertolazzo A. A.; Molinero V. How Do Surfactants Control the Agglomeration of Clathrate Hydrates?. ACS Cent. Sci. 2019, 5, 428–439. 10.1021/acscentsci.8b00755. 30937370 PMC6439454

[ref12] Anderson B. J.; Tester J. W.; Borghi G. P.; Trout B. L. Properties of Inhibitors of Methane Hydrate Formation Via Molecular Dynamics Simulations. J. Am. Chem. Soc. 2005, 127, 17852–17862. 10.1021/ja0554965. 16351116

[ref13] Bui T.; Phan A.; Monteiro D.; Lan Q.; Ceglio M.; Acosta E.; Krishnamurthy P.; StrioloD A. Evidence of Structure-Performance Relation for Surfactants Used as Antiagglomerants for Hydrate Management. Langmuir 2017, 33, 2263–2274. 10.1021/acs.langmuir.6b04334. 28110536

[ref14] Zerpa L. E.; Salager J. L.; Koh C. A.; Sloan E. D.; Sum A. K. Surface Chemistry and Gas Hydrates in Flow Assurance. Ind. Eng. Chem. Res. 2011, 50, 188–197. 10.1021/ie100873k.

[ref15] Phan A.; Stamatakis M.; Koh C. A.; Striolo A. Correlating Antiagglomerant Performance with Gas Hydrate Cohesion. ACS Appl. Mater. Interfaces 2021, 13, 40002–40012. 10.1021/acsami.1c06309. 34382786

[ref16] Phan A.; Stamatakis M.; Koh C. A.; Striolo A. Wetting Properties of Clathrate Hydrates in the Presence of Polycyclic Aromatic Compounds: Evidence of Ion-Specific Effects. J. Phys. Chem. Lett. 2022, 13, 8200–8206. 10.1021/acs.jpclett.2c01846. 36006399 PMC9442800

[ref17] Phan A.; Stoner H. M.; Stamatakis M.; Koh C. A.; Striolo A. Surface Morphology Effects on Clathrate Hydrate Wettability. J. Colloid Interface Sci. 2022, 611, 421–431. 10.1016/j.jcis.2021.12.083. 34968961

[ref18] Hassanpouryouzband A.; Joonaki E.; Vasheghani Farahani M.; et al. Gas Hydrates in Sustainable Chemistry. Chem. Soc. Rev. 2020, 49, 5225–5309. 10.1039/C8CS00989A. 32567615

[ref19] Babu P.; Nambiar A.; He T. B.; Karimi I. A.; Lee J. D.; Englezos P.; Linga P. A Review of Clathrate Hydrate Based Desalination to Strengthen Energy-Water Nexus. ACS Sustainable Chem. Eng. 2018, 6, 8093–8107. 10.1021/acssuschemeng.8b01616.

[ref20] Papadimitriou N. I.; Tsimpanogiannis I. N.; Economou I. G.; Stubos A. K. Monte Carlo Simulations of the Separation of a Binary Gas Mixture (CH4 + CO2) Using Hydrates. Phys. Chem. Chem. Phys. 2018, 20, 28026–28038. 10.1039/C8CP02050G. 30383048

[ref21] Pang J.; Liang Y.; Masuda Y.; Takeya S. Structural Transition of the Methane–Ethane Mixture Hydrate in a Hydrate/Water/Hydrocarbon Three-Phase Coexistence System: Effect of Gas Concentration. ACS Sustainable Chem. Eng. 2020, 8, 16924–16937. 10.1021/acssuschemeng.0c06432.

[ref22] Nguyen N. N.; Nguyen C. V.; Nguyen T. A. H.; Nguyen A. V. Surface Science in the Research and Development of Hydrate-Based Sustainable Technologies. ACS Sustainable Chem. Eng. 2022, 10, 4041–4058. 10.1021/acssuschemeng.2c00028.

[ref23] Khurana M.; Veluswamy H. P.; Daraboina N.; Linga P. Thermodynamic and Kinetic Modelling of Mixed Ch4-Thf Hydrate for Methane Storage Application. Chem. Eng. J. 2019, 370, 760–771. 10.1016/j.cej.2019.03.172.

[ref24] Sugahara T.; Haag J. C.; Prasad P. S. R.; Warntjes A. A.; Sloan E. D.; Sum A. K.; Koh C. A. Increasing Hydrogen Storage Capacity Using Tetrahydrofuran. J. Am. Chem. Soc. 2009, 131, 14616–14617. 10.1021/ja905819z. 19780560

[ref25] Phan A.; Schlösser H.; Striolo A. Molecular Mechanisms by Which Tetrahydrofuran Affects Co2 Hydrate Growth: Implications for Carbon Storage. Chem. Eng. J. 2021, 418, 129423–129433. 10.1016/j.cej.2021.129423.

[ref26] Takeya S.; Ripmeester J. A. Dissociation Behavior of Clathrate Hydrates to Ice and Dependence on Guest Molecules. Angew. Chem., Int. Ed. 2008, 47, 1276–1279. 10.1002/anie.200703718. 18183557

[ref27] Torré J.-P.; Ricaurte M.; Dicharry C.; Broseta D. Co2 Enclathration in the Presence of Water-Soluble Hydrate Promoters: Hydrate Phase Equilibria and Kinetic Studies in Quiescent Conditions. Chem. Eng. Sci. 2012, 82, 1–13. 10.1016/j.ces.2012.07.025.

[ref28] Veluswamy H. P.; Premasinghe K. P.; Linga P. Co2 Hydrates – Effect of Additives and Operating Conditions on the Morphology and Hydrate Growth. Energy Procedia 2017, 105, 5048–5054. 10.1016/j.egypro.2017.03.1019.

[ref29] Junthong S.; Yodpetch V.; Inkong K.; Kulprathipanja S.; Rangsunvigit P. Effects of Pyrrolidine on Methane Hydrate Formation: Thermodynamic, Kinetic, and Morphology Perspectives. J. Nat. Gas Sci. Eng. 2021, 96, 104322–104333. 10.1016/j.jngse.2021.104322.

[ref30] Lee B.; Shin K.; Muromachi S.; Moudrakovski I. L.; Ratcliffe C. I.; Ripmeester J. A. Enhanced Methane Storage in Clathrate Hydrates Induced by Antifreezes. Chem. Eng. J. 2021, 418, 129304–129311. 10.1016/j.cej.2021.129304.

[ref31] Gabriel S. Ueber Vinylamin. Ber. Dtsch. Chem. Ges. 1888, 21, 1049–1057. 10.1002/cber.188802101196.

[ref32] Piou T.; Campeau L.-C. Bringing Amines Back into Aziridination. Nat. Chem. 2021, 13, 1027–1028. 10.1038/s41557-021-00819-7. 34697398

[ref33] Degennaro L.; Trinchera P.; Luisi R. Recent Advances in the Stereoselective Synthesis of Aziridines. Chem. Rev. 2014, 114, 7881–7929. 10.1021/cr400553c. 24823261

[ref34] Míguez J. M.; Conde M. M.; Torre J. P.; Blas F. J.; Pineiro M. M.; Vega C. Molecular Dynamics Simulation of Co2 Hydrates: Prediction of Three Phase Coexistence Line. J. Chem. Phys. 2015, 142, 124505–124516. 10.1063/1.4916119. 25833594

[ref35] Yagasaki T.; Matsumoto M.; Tanaka H. Formation of Clathrate Hydrates of Water-Soluble Guest Molecules. J. Phys. Chem. C 2016, 120, 21512–21521. 10.1021/acs.jpcc.6b06498.

[ref36] Zhang Y.; Maginn E. J. A Comparison of Methods for Melting Point Calculation Using Molecular Dynamics Simulations. J. Chem. Phys. 2012, 136, 144116–144127. 10.1063/1.3702587. 22502510

[ref37] Takeuchi F.; Hiratsuka M.; Ohmura R.; Alavi S.; Sum A. K.; Yasuoka K. Water Proton Configurations in Structures I, Ii, and H Clathrate Hydrate Unit Cells. J. Chem. Phys. 2013, 138, 124504–124515. 10.1063/1.4795499. 23556733

[ref38] Delahaye A.; Fournaison L.; Marinhas S.; Chatti I.; Petitet J. P.; Dalmazzone D.; Furst W. Effect of Thf on Equilibrium Pressure and Dissociation Enthalpy of Co2 Hydrates Applied to Secondary Refrigeration. Ind. Eng. Chem. Res. 2006, 45, 391–397. 10.1021/ie050356p.

[ref39] Lee Y. J.; Kawamura T.; Yamamoto Y.; Yoon J. H. Phase Equilibrium Studies of Tetrahydrofuran (Thf) + Ch4, Thf + Co2, Ch4 + Co2, and Thf + Co2 + Ch4 Hydrates. J. Chem. Eng. Data 2012, 57, 3543–3548. 10.1021/je300850q.

[ref40] Jensen L.; Thomsen K.; von Solms N.; Wierzchowski S.; Walsh M. R.; Koh C. A.; Sloan E. D.; Wu D. T.; Sum A. K. Calculation of Liquid Water-Hydrate-Methane Vapor Phase Equilibria from Molecular Simulations. J. Phys. Chem. B 2010, 114, 5775–5782. 10.1021/jp911032q. 20392117

[ref41] Walsh M. R.; Koh C. A.; Sloan E. D.; Sum A. K.; Wu D. T. Microsecond Simulations of Spontaneous Methane Hydrate Nucleation and Growth. Science 2009, 326, 1095–1098. 10.1126/science.1174010. 19815725

[ref42] Abascal J. L. F.; Sanz E.; Fernandez R. G.; Vega C. A Potential Model for the Study of Ices and Amorphous Water: Tip4p/Ice. J. Chem. Phys. 2005, 122, 234511–234519. 10.1063/1.1931662. 16008466

[ref43] Harris J. G.; Yung K. H. Carbon Dioxide’s Liquid-Vapor Coexistence Curve and Critical Properties as Predicted by a Simple Molecular Model. J. Phys. Chem. A 1995, 99, 12021–12024. 10.1021/j100031a034.

[ref44] Tung Y. T.; Chen L. J.; Chen Y. P.; Lin S. T. Growth of Structure I Carbon Dioxide Hydrate from Molecular Dynamics Simulations. J. Phys. Chem. C 2011, 115, 7504–7515. 10.1021/jp112205x. 20669917

[ref45] Wang J. M.; Wolf R. M.; Caldwell J. W.; Kollman P. A.; Case D. A. Development and Testing of a General Amber Force Field. J. Comput. Chem. 2004, 25, 1157–1174. 10.1002/jcc.20035. 15116359

[ref46] Case D. A. Amber 14; University of California: San Francisco, 2014.

[ref47] Eastwood J. W.; Hockney R. W.; Lawrence D. N. P3m3dp - the 3-Dimensional Periodic Particle-Particle-Particle-Mesh Program. Comput. Phys. Commun. 1980, 19, 215–261. 10.1016/0010-4655(80)90052-1.

[ref48] Allen M. P.; Tildesley D. J. Computer Simulation of Liquids; Oxford University Press: Oxford, U.K., 2004.

[ref49] Abraham M. J.; Murtola T.; Schulz R.; Páll S.; Smith J. C.; Hess B.; Lindahl E. Gromacs: High Performance Molecular Simulations through Multi-Level Parallelism from Laptops to Supercomputers. SoftwareX 2015, 1–2, 19–25. 10.1016/j.softx.2015.06.001.

[ref50] Yagasaki T.; Matsumoto M.; Tanaka H. Mechanism of Slow Crystal Growth of Tetrahydrofuran Clathrate Hydrate. J. Phys. Chem. C 2016, 120, 3305–3313. 10.1021/acs.jpcc.5b10293.

[ref51] Kästner J. Umbrella Sampling. WIREs Comput. Mol. Sci. 2011, 1, 932–942. 10.1002/wcms.66.

[ref52] Hub J. S.; de Groot B. L.; van der Spoel D. G_Wham-a Free Weighted Histogram Analysis Implementation Including Robust Error and Autocorrelation Estimates. J. Chem. Theory Comput. 2010, 6, 3713–3720. 10.1021/ct100494z.

[ref53] Mahmoudinobar F.; Dias C. L. Grade: A Code to Determine Clathrate Hydrate Structures. Comput. Phys. Commun. 2019, 244, 385–391. 10.1016/j.cpc.2019.06.004.

[ref54] Adisasmito S.; Frank R. J.; Sloan E. D. Hydrates of Carbon-Dioxide and Methane Mixtures. J. Chem. Eng. Data 1991, 36, 68–71. 10.1021/je00001a020.

[ref55] Dybeck E. C.; Abraham N. S.; Schieber N. P.; Shirts M. R. Capturing Entropic Contributions to Temperature-Mediated Polymorphic Transformations through Molecular Modeling. Cryst. Growth Des. 2017, 17, 1775–1787. 10.1021/acs.cgd.6b01762.

[ref56] Mobley D. L.; Klimovich P. V. Perspective: Alchemical Free Energy Calculations for Drug Discovery. J. Chem. Phys. 2012, 137, 230901–230912. 10.1063/1.4769292. 23267463 PMC3537745

[ref57] Tohidi B.; Danesh A.; Todd A. C.; Burgass R. W.; Østergaard K. K. Equilibrium Data and Thermodynamic Modelling of Cyclopentane and Neopentane Hydrates. Fluid Phase Equilib. 1997, 138, 241–250. 10.1016/S0378-3812(97)00164-7.

[ref58] Ismail N. A.; Koh C. A. Growth Rate and Morphology Study of Tetrahydrofuran Hydrate Single Crystals and the Effect of Salt. CrystEngComm 2022, 24, 4301–4311. 10.1039/D2CE00176D.

[ref59] Shin W.; Park S.; Ro H.; Koh D.-Y.; Seol J.; Lee H. Phase Equilibrium Measurements and the Tuning Behavior of New Sii Clathrate Hydrates. J. Chem. Thermodyn. 2012, 44, 20–25. 10.1016/j.jct.2011.08.018.

[ref60] Dobrzycki L.; Taraszewska P.; Boese R.; Cyrański M. K. Pyrrolidine and Its Hydrates in the Solid State. Cryst. Growth Des. 2015, 15, 4804–4812. 10.1021/acs.cgd.5b00527.

[ref61] Shultz M. J.; Vu T. H. Hydrogen Bonding between Water and Tetrahydrofuran Relevant to Clathrate Formation. J. Phys. Chem. B 2015, 119, 9167–9172. 10.1021/jp509343x. 25427311

[ref62] Yagasaki T.; Matsumoto M.; Tanaka H. Adsorption Mechanism of Inhibitor and Guest Molecules on the Surface of Gas Hydrates. J. Am. Chem. Soc. 2015, 137, 12079–12085. 10.1021/jacs.5b07417. 26331549

[ref63] Bui T.; Sicard F.; Monteiro D.; Lan Q.; Ceglio M.; Burress C.; Striolo A. Antiagglomerants Affect Gas Hydrate Growth. J. Phys. Chem. Lett. 2018, 9, 3491–3496. 10.1021/acs.jpclett.8b01180. 29870264

[ref64] Moudrakovski I. L.; Udachin K. A.; Alavi S.; Ratcliffe C. I.; Ripmeester J. A. Facilitating Guest Transport in Clathrate Hydrates by Tuning Guest-Host Interactions. J. Chem. Phys. 2015, 142, 074705–074714. 10.1063/1.4907720. 25702022

[ref65] Heinz H. Computational Screening of Biomolecular Adsorption and Self-Assembly on Nanoscale Surfaces. J. Comput. Chem. 2010, 31, 1564–1568. 10.1002/jcc.21421. 19862812

[ref66] Emami F. S.; Puddu V.; Berry R. J.; Varshney V.; Patwardhan S. V.; Perry C. C.; Heinz H. Prediction of Specific Biomolecule Adsorption on Silica Surfaces as a Function of Ph and Particle Size. Chem. Mater. 2014, 26, 5725–5734. 10.1021/cm5026987.

[ref67] Hudait A.; Qiu Y.; Odendahl N.; Molinero V. Hydrogen-Bonding and Hydrophobic Groups Contribute Equally to the Binding of Hyperactive Antifreeze and Ice-Nucleating Proteins to Ice. J. Am. Chem. Soc. 2019, 141, 7887–7898. 10.1021/jacs.9b02248. 31020830

